# Implementing is necessary: the gap between discourse and practice in global open science

**DOI:** 10.1590/1807-3107bor-2026.vol40.013

**Published:** 2026-05-08

**Authors:** Maíra Terra GARCIA, Fabiana Alves de Souza SILVA, Maria Eduarda da Silva COSTA, Paulo Henrique Fonseca do CARMO, Patricia Michelle Nagai de LIMA, Lara Luise Castro PEDROSO, Fernanda Alves FEITOSA, Sigmar de Mello RODE

**Affiliations:** (a) Universidade Estadual Paulista – Unesp, Institute of Science and Technology, Department of Oral Science and Diagnosis, São José dos Campos, Brazil; São José dos Campos, SP, Brazil.; (b) Universidade Estadual Paulista – Unesp, Institute of Biosciences, Department of Genetics, Microbiology and Immunology, Botucatu, SP, Brazil.

**Keywords:** Open Science, Public Policy, Barriers to Open Science

## Abstract

Open science aims to promote more transparent, collaborative, and accessible practices in the scientific process. However, barriers such as fear of exposure or reluctance to share data and methods make researchers hesitant to amply engage with this movement. This study analyzes the global open science movement and the persistent gap between its discourse and practice. Based on a critical narrative review of historical milestones, the analysis demonstrates that although open science promotes transparency, collaboration, reproducibility, and stronger connections among science, society, and policy, its adoption remains uneven and limited. Individual barriers, such as a lack of training and fear of misuse, along with institutional obstacles related to publishing models, evaluation metrics, and financial constraints, hinder the full implementation of open practices. Contrastingly, significant benefits are evident, including cost reduction, acceleration of scientific progress through data reuse, and reinforcement of evidence-based public policies. Worldwide experiences highlight both the advances and inequalities in access and infrastructure, demonstrating that the global impact of open science depends on more equitable participation. In conclusion, bridging the gap between discourse and practice requires structural changes in research evaluations, cultural shifts that prioritize collective knowledge, and stronger integration between researchers, policymakers, and society. Implementing open science is essential to ensure that knowledge production truly serves global development and social equity.

## What is open science?

Open science is a movement that promotes transparent, collaborative, and accessible practices in the scientific process with the goal of democratizing knowledge and accelerating scientific progress. This term refers to practices that make scientific knowledge more accessible and encourage cooperation among researchers worldwide.^1^ This change has social and cultural dimensions and proposes a new way of conducting, understanding, and accessing scientific research.^2^ The ongoing commitment to open science involves fostering an environment in which knowledge is shared equitably and applied for the common good.^3^

Pinpointing the exact beginning of this movement is difficult; however, it has clearly been gaining traction over the decades. Some historical milestones can be highlighted, such as the report Science, The Endless Frontier (1945) by Vannevar Bush, which emphasized the importance of disseminating scientific knowledge for the public good.^4^ The “journal crisis” of the 1990s was also an essential milestone in the history of open science. It resulted from a sharp increase in subscription prices for scientific journals, which drastically affected libraries, specifically in the United States. This price escalation made it unfeasible for many academic institutions to maintain complete collections, creating a gap in access to knowledge.^5^ This scenario contributed to the emergence of the first open-access journals, which remain the most well-known pillars of open science today.

In 1996, the Scientific Electronic Library Online (SciELO) was created in Brazil and became a pioneer in open access to scientific journals. In 2002, the landmark Budapest Open Access Initiative (BOAI), a declaration of principles aimed at promoting free and unrestricted access to academic and scientific content with the goal of democratizing knowledge and overcoming payment barriers that limited access to quality research, took place.^6^ In 2005, the Brazilian Manifesto in Support of Open Access to Scientific Information was launched, positioning Brazil as an active participant in this movement.^7^ More recently, in 2021, the United Nations Educational, Scientific and Cultural Organization (UNESCO) emphasized the importance of open science, stating that it is a “vital tool to improve the quality and accessibility of both scientific results and the scientific process”.^2^

Following this global trend, Plan S, an initiative launched in 2021 by European funding agencies, established mandatory guidelines for the publication of research funded by national, regional, and international councils and agencies, both public and private. Among these guidelines is the requirement that research be published in open-access journals or platforms and that results be made immediately available in open repositories (https://www.coalition-s.org/). In the Brazilian context, in 2023, the SciELO digital library celebrated 25 years of open-access operations, reaffirming its commitment to principles such as open science at all stages of the research process, combating predatory editorial practices, and promoting inclusion and diversity on editorial boards.^8^

There are various definitions of open science, as it encompasses a wide range of practices, including open access to publications, open research data, open-source software and tools, open workflows, citizen science, open educational resources, and alternative methods of research evaluation such as open peer review ^9^. All these practices are considered pillars of open science and are closely interconnected with the FAIR principles —Findable, Accessible, Interoperable, and Reusable^10,11^—introduced by Wilkinson et al. in 2016 through the FAIR Guiding Principles.^12^ They complement each other in building more transparent, collaborative, and accessible scientific practices. While the pillars of open science set core values, the FAIR principles provide practical guidelines for applying these values in everyday scientific research^13^ For example, in the context of open data, the FAIR principles ensure that openness is effective, practical, and secure. Data made available in a disorganized manner or without clear metadata may be technically open but not useful.^14^

Another crucial pillar that the open science movement has been advancing is open peer review. Traditional peer review, a practice widely used in scientific journals, involves the selection of experts in the field to evaluate and ensure the quality, relevance, and originality of a work prior to its publication.^8^ However, this practice has revealed numerous problems, the most significant of which is the lack of transparency regarding who reviews the articles. Predatory journals have taken advantage of this anonymity to accept articles with superficial reviews or even without any evaluation. Such practices greatly harm the credibility of global science.

However, the term open peer review has different interpretations, depending on the context and perspective adopted. For some, it refers to a process in which the identities of authors and reviewers are mutually known, thereby promoting greater transparency. Others associate it with the practice of publishing reviewer reports alongside articles, allowing readers to access the evaluation process. From a broader perspective, the term can encompass both approaches or even systems that enable comments from other members of the scientific community in addition to invited reviewers. Some use the concept to describe various combinations of these practices or the inclusion of new methodologies aimed at making the evaluation process more inclusive, collaborative, and transparent.^15^ Regardless of the specific definition, the central objective of open peer review is to strengthen the evaluation process by improving integrity, trust, and the quality of scientific communication while facilitating constructive dialogue among researchers and reviewers and promoting more accessible and democratic science.^16^

## The role of science in public policy

Open science is crucial in strengthening the interface between science and policy, and in reshaping how scientific knowledge is produced, shared, and applied in public decision-making processes. When effectively implemented, this interaction between science and politics offers numerous benefits to society, fostering an environment in which public decisions are grounded in robust evidence and are aimed at promoting collective well-being.^17^ By fostering dialogue between politics and science, public policies can become more efficient, transparent, and aligned with society’s real needs.

Overall, however, a gap remains between scientific evidence and political decision-making. This gap arises when scientific results are not adequately incorporated into political processes, whether owing to limited access, insufficient understanding, or lack of acceptance by decision-makers, often stemming from structural, cultural, or institutional barriers that hinder integration.^18^ The mere availability of scientific evidence alone is insufficient to guarantee its use in political processes.

Therefore, open science must be accompanied by targeted efforts to improve the communication and interaction between researchers and government ministries. Through more effective communication and the provision of data and evidence, science can serve as an essential pillar for the development of public policies that are more efficient, fair, and aligned with social and environmental needs.^19^

The adoption of public policies based on scientific evidence ensures that government decisions rely on solid information, thereby promoting transparency and effectiveness. Health, climate change, and economic development are examples of areas in which science has been instrumental in guiding high-quality interventions and programs.

In this context, scientific dissemination plays a strategic role in linking scientific knowledge with society. This involves translating complex findings into an accessible language, enabling the public to understand and support policy decisions ^17^. Open science promotes the use of scientific evidence to inform and guide policy-making, emphasizing that policy choices should be based on data rather than ideology or personal beliefs.^20^

Furthermore, it is essential that science returns the knowledge generated by publicly funded research to society as this reinforces the transparency, equity, and social impact of scientific endeavors. When society invests resources in research, either directly or through taxes, it expects the results not only to advance knowledge but also to contribute to solving practical problems, improving the quality of life, and promoting sustainable development.

For example, in 2020, during the COVID-19 pandemic, the advantages of open communication became evident. The publication of preprints, scientific articles with open access, and the dissemination of materials in accessible language facilitated the greater involvement of society, government authorities, health professionals, and media. This access stimulated broad discussions at all levels, reinforcing the importance of science in society and enhancing the benefits obtained from the results of scientific research.

As important interlocutors between science and the community, the inclusion of care professionals, such as workers from the SUS and private health institutions, is essential.^21^ These professionals can not only benefit from open science through access to reliable sources of information for reading and updating, but also be part of research, contributing with their experiences and practical experiences in the field.

Therefore, citizen science relies on the dissemination of high-quality information. This approach involves public participation in scientific initiatives, allowing interested individuals to contribute to the advancement of scientific knowledge while generating knowledge for the participants themselves.^22^ Although their involvement is often associated with data collection, it can also extend to planning and participation at various stages of a project. Such engagement increases public awareness of science, fosters community interest, and promotes a society capable of developing scientific thinking based on evidence, ultimately helping combat misinformation and the spread of fake news.

The Brazilian Biodiversity Information System (SIBBr) is a notable example of the open science movement as it provides scientific information that is accessible and free of charge to all interested parties; promotes collaboration among scientists, citizens, and institutions; offers reliable data; and engages citizens in the collection and organization of information, thereby expanding the reach of research and fostering scientific literacy. Through these measures, the project successfully fulfills its objectives of integrating, organizing, and making information on Brazilian biodiversity widely available.

## Barrier to open science

The open science movement seeks to promote equity in scientific knowledge by fostering transparency and aims to dismantle socioeconomic and cultural barriers^23,24^ Within the scientific community, there is often a distorted perception that “true” knowledge is produced by developed countries or that only publications in high-impact journals are genuinely reliable. This view implies that research conducted and published outside this scope does not deserve universal recognition, thereby devaluing the contributions of underdeveloped countries and their national journals^25^

Building on the barriers discussed in the previous section, it is evident that for the effective implementation of open science in national public policies, publications that incorporate premises and actions aligned with the Brazilian Constitution and public policies are essential. Such studies hold greater practical relevance than those published in major international journals, which, although prestigious, often lack a connection to Brazil’s social, developmental, and health contexts.

Despite the great potential of open science to accelerate academic progress, whether through open access or data sharing, its adoption remains limited, largely because of the persistence of individual and institutional barriers^24,26^

Regarding individual barriers, many researchers are not familiar with open science practices such as data sharing, open access, and collaborative platforms, owing to a lack of knowledge, training, and incentives. Consequently, the potential of open science remains underutilized^1,27^ When confronted with the concept of open science, many researchers tend to associate it solely with open access to journals without realizing that it encompasses a wide range of practices. They are often unaware of the benefits that openness can provide, such as accelerating scientific progress and reducing both the time and cost of research development^1,28^

Furthermore, many researchers do not know how or where to share their data, nor do they recognize the potential benefits of doing so for their own work. This lack of knowledge regarding how to adopt open science practices contributes to low adherence to the movement^1,29,30^

Reluctance and fear are among the main barriers to the wider adoption of open science because self-interest often influences decisions to share data or methods. Many researchers believe that it is unfair for others to benefit from data generated through their own labor. However, this perspective overlooks the fact that science is conducted for the collective good of advancing society as a whole rather than for individual achievement alone. Another significant barrier is the fear of being exposed to improper practices or that alternative analyses of their data might reveal invalid conclusions^29^

Laine^31^ highlights that the greatest concern researchers face when sharing data is the risk of scooping—the possibility that others might claim priority over their ideas or results, presenting themselves as the first to conduct a given study or even appropriating the data. This issue is compounded by the widespread belief that academic journals value innovation and positive results. Moreover, researchers emphasize that factors such as data protection policies, trust in the systems being implemented, and the preservation of academic autonomy can foster resistance or hesitation toward adopting open science practices.^32^

Regarding institutional barriers, academic culture uses journal publications as metrics for evaluating research and researchers. Consequently, there is pressure, both internally and externally, for high publication output in journals, which leads many to follow the motto “publish or perish”.^31^ However, the high costs of publication in open-access journals make it difficult for researchers who do not have financial support to meet established metrics, thereby hindering researchers from developing countries from succeeding in open-access publishing.^30^

Overcoming these barriers requires collective, individual, institutional, and even business efforts. The monopolization of knowledge should not be encouraged. Initiatives that motivate researchers to adhere to open science practices, such as changing evaluation and funding metrics along with training, are fundamental to changing the current scenario.^30^

## Benefits of open science

Considering that the concept of open science is broad, encompassing the entire process of scientific production from planning to publication, there are numerous benefits that accompany it.^1^

Practices associated with open science enable scientific knowledge to become more accessible to individuals within the academic field, especially those from countries or institutions with limited resources, thus allowing for greater reproducibility. Additionally, open science policies promote greater interdisciplinary and international collaboration, breaking down geographical and sectoral barriers. Consequently, this generates greater reliability in the data presented and supports the dynamic nature of science, which constantly renews and reinvents itself to produce knowledge.^13^

In addition to promoting access to articles, open science provides financial benefits for global research. For example, the reuse of open data and resources reduces the duplication of research. When data from previous studies are made available in an accessible form and in compliance with the FAIR principles,^12^ other researchers can use them as a basis for new studies, saving time and resources that would otherwise be needed to generate similar data.^33^ The registration of research in repositories is also an important approach within open science; this also has economic impacts, as it makes knowledge available free of charge, prevents the publication of very similar studies, and consequently reduces expenses by avoiding repetition. It also allows resources to be redirected toward new research stages or the advancement of other studies.^34,35^

When the focus is on all the advantages of open science, it becomes evident that denying these practices can have serious consequences. The main limitation is that restricted access to scientific literature directly affects researchers’ communication with the public. By not allowing information to be obtained easily and transparently, research becomes less clear and reliable, social engagement with science is reduced, and serious debates arise about the reliability of the knowledge presented.^36^

## Toward an open scientific community: perspectives and challenges

Global efforts are increasingly focused on creating a genuinely open scientific community. Open science initiatives, advancing at different paces worldwide, are driven by a combination of national policies and regional collaborations.^37-39^

Since 2012, the European Commission has actively promoted an open model of science and research, encouraging Member States to develop and implement national strategies. Currently, Horizon Europe 2021–2027 requires all beneficiaries to ensure open access to peer-reviewed academic publications, which must be available online and free of charge to the public. This can be achieved through a repository (green open access), the publisher’s website (mostly gold open access), or a publishing platform.^40^ This program is also moving forward with a mandatory data management plan and continues to support the development of the European Open Science Cloud (EOSC), a federated technology infrastructure that allows European scientists and researchers to share and process research data.^41^

The European Commission requires that research data be “as open as possible and as closed as necessary.” Along with open access to research information and data, the program emphasizes the development of state-of-the-art metrics, incorporating open science into scientific assessments, advocating for common standards of research integrity, and promoting education and citizen science.^42^ European countries and other nations associated with the Horizon program are part of the European Research Area, a collaborative initiative that aims to foster a cohesive research and innovation ecosystem across Europe (EU, 2021 – https://eur-lex.europa.eu/eli/reco/2021/2122/oj). Indeed, the efforts of the European community are often supported by international organizations such as the United Nations Educational, Scientific and Cultural Organization (UNESCO) and regional initiatives that encourage cross-border cooperation in open data sharing and scientific collaboration.^43,44^

In Asia, open science has seen significant advancements, with several countries establishing policies and infrastructure to promote open access to research.^45^ For example, Japan launched the Science Information Network (SINET) to support the sharing of academic and research data between universities and research institutions (SINET 6, 2024 – https://www.sinet.ad.jp/en). The country has been promoting the concept of open science since 2015 with a national open science policy that emphasizes open access to publicly funded research data.^9^

China has also been active in open science, particularly through the China Science and Technology Cloud, which provides a centralized platform for sharing and storing data among researchers (https://goscloud.net/serviceDetail/64f98bbaa4303c0a65981bfb). South Korea supports open science through the Korea Open Science and Technology Information Center, which provides access to publications and research data. The Korean government promotes open science as part of its broader innovation and technological development strategy, encouraging collaboration between public and private research institutions (https://www.msit.go.kr/eng/index.do). Other countries, such as India, Singapore, and Malaysia, are also taking steps to promote open science through government-backed initiatives, open access repositories, and policies aimed at increasing the accessibility and reproducibility of research. Moreover, the Open-Asia Project, launched in January 2024 as a collaboration between nine partner organizations from Europe and Asia, aims to promote the digital economy and connectivity in India and Malaysia by promoting the principles and values of open science (https://openasia-erasmus.org/ – https://openscience.um.edu.my/open-asia-erasmus-cbhe).

In Oceania, the status of open science is evolving, with several key initiatives promoting the concept. The region’s open science efforts are gaining momentum, particularly in countries such as Australia, which has long supported open data initiatives.^46^ A significant aspect of open science in Oceania is the push for regional cooperation and establishment of platforms that can integrate resources between countries (https://www.caul.edu.au/services-programs/australian-open-science-network). Along with Australia, New Zealand plays a crucial role in driving open science in the Pacific region, developing strategies to improve data access and fostering collaborations with regional countries. Australia has been working in accordance with the UNESCO, the Organisation for Economic Co-operation and Development (OECD), and the Asia-Pacific Economic Cooperation (APEC) open science declaration (https://oaaustralasia.org/events/open-science-in-australia-how-can-we-support-key-international-initiatives/). This collaboration aims to establish shared data infrastructures, improve synergies between science and policy, and promote data-driven solutions that align with scientific and socioeconomic priorities. Additionally, these partnerships have improved the integrity, reliability, and transparency of scientific research and have facilitated the flow of knowledge between universities and research institutes for the government, society, and industry.^46^

The United States designated 2023 as the “Year of Open Science,” led by the White House Office of Science and Technology Policy (OSTP). This initiative brought together federal agencies with a diverse range of communities, including students, researchers, universities, private companies, libraries, and foundations, to ensure that science and technology benefit communities equitably across the country. The goals of the OSTP included promoting free, immediate, and equitable access to federally funded research, strengthening Open Science policies, investing in Open Science infrastructure, and supporting the research community in building Open Science capacities. Additionally, the initiative sought to expand participation in Open Science and encourage open research practices.

Several national agencies, including the Administration for Community Life, Agency for Health Care Research and Quality, Department of Energy, National Aeronautics and Space Administration (NASA), National Science Foundation (NSF), National Institutes of Health (NIH), and National Institute of Standards and Technology (NIST), have voluntarily published updated or newly developed Open Science plans. These plans, aligned with the OSTP guidelines, are intended to enhance community engagement and support the development of Open Science policies in 2023 (https://fas.org/accelerator/open-science/).

In North America, Canadian agencies have been strong advocates for making the research results they fund as accessible as possible. Through the Chief Science Advisor to Canada’s Roadmap to Open Science, the government is currently advocating for the development of strategies to ensure that departmental science data is FAIR (Findable, Accessible, Interoperable, and Reusable). This effort ensures quality data management and improves public access to research^47^ (https://www.canada.ca/en/environment-climate-change/services/science-technology/open-science-action-plan.html#toc1).

In Africa, investment in open science led to the establishment of the African Open Science Platform (AOSP) in 2017, which created a federated infrastructure comprising hardware, communications, and software, along with supporting policies and practices for open data initiatives. This effort includes organizations such as AfricArXiv and is supported by agencies such as the South African Department of Science and Innovation, International Science Council, South African Academy of Science, Open Research Africa, and several national research facilities (https://info.africarxiv.org/about/). Furthermore, the East African Commission on Science and Technology collaborates with international entities to promote open science policies among its member states with the aim of increasing transparency and access to research data.^48^ Currently, AOSP focuses on promoting the production of new knowledge and the development of qualified researchers. These include exploring innovative methods for public engagement with research, launching grant programs to drive systemic change, and establishing training and skill development initiatives for high-quality research engagement and evaluation^45^ https://www.rd-alliance.org/wp-content/uploads/2024/05/AOSP-Briefing.pdf.

Since the late 20^th^ century, Latin America has made substantial contributions to the advancement of open science even before this topic gained attention in other regions.^45^ Initiatives such as the Scientific Electronic Library Online (SciELO) created in 1996 and the scientific information system Redalyc created in 2003 have promoted open science by providing open access to scientific articles in numerous journals^49^. More recently, the impact of these databases has expanded with the establishment of national Current Research Information Systems (CRIS) such as BrCRIS and PeruCRIS (https://perucris.concytec.gob.pe/). These platforms consolidate and unify information about researchers, institutions, repositories, open datasets, research projects, and citizen scientists.^50^ In this context, the move toward open science, which began with access to publications, has now advanced to include open research data.^21,51,52^.

Several Latin American countries have strengthened their commitment to open science through national and regional strategies that prioritize inclusion, accessibility, and citizen participation. In Brazil, the National Open Science Policy is coordinated by the Brazilian Institute of Information in Science and Technology (IBICT), which promotes open access repositories, data management standards, and the integration of scientific information through systems, such as the Brazilian Biodiversity Information System (SiBBr) and BrCRIS. The National Consortium for Open Science (CoNCienciA network) and SciELO Brazil are central to promoting scientific transparency and dissemination in the region. In Mexico, initiatives such as the National Consortium of Scientific and Technological Information Resources (CONRICyT) and the National Repository (Repositorio Nacional) have expanded access to national research outputs. Argentina has implemented the Sistema Nacional de Repositorios Digitales (SNRD), a pioneering framework for open data and publications. Colombia’s Ministry of Science, Technology, and Innovation (MinCiencias) has supported the creation of institutional repositories and citizen science programs, whereas Ecuador has launched the Abierta EC initiative to promote open data in education and environmental sciences. Collectively, these efforts illustrate the region’s progress in aligning with the UNESCO’s Recommendation on Open Science, emphasizing collaboration, multilingual accessibility, and the reduction of inequalities in scientific communication.^22,44^

The global impact of open science has been demonstrated through landmark initiatives such as the Human Genome Project, Symbiota, and Global Open Data for Agriculture and Nutrition (National Academies of Sciences, Engineering, and Medicine; Politics and Global Affairs; Research Data and Information Council; Committee on the Development of a Toolkit to Promote Open Science Practices: a workshop; Kameyama E, Saunders J, Arrison T, editors. Washington: National Academies Press; 2021). More recently, open access to research data, resources, and methodologies has facilitated a rapid global response to the COVID-19 pandemic. Despite this progress, this period also underscored the need to expand open science practices across research sectors, as the extensive use of preprints during the pandemic led to premature, non-peer-reviewed findings being widely reported in the media.^39,53,54^

Although many federal departments have implemented policies and initiatives to promote open science, challenges persist, including high publication rates, limited funding, predatory journal behavior, and infrastructure gaps. For example, in underdeveloped and developing countries, infrastructure limitations, especially in relation to internet connectivity, pose significant challenges to the implementation of open science. Limited internet access remains a barrier to accessing open science resources in certain regions^48,55^ Additionally, the absence of uniform policies among nations and the need for capacity building in open science practices are prevalent obstacles. Cultural factors, such as lack of incentives, limited knowledge of open data access portals, and limited understanding of data sharing strategies, have also impeded the progress of open science. Despite these challenges, efforts to promote open science are crucial to promote scientific transparency, collaboration, and global access to data.^37,39,56^

## Data Availability

The authors declare that all data generated or analyzed during this study are included in this published article.
